# ATRA modulates mechanical activation of TGF-β by pancreatic stellate cells

**DOI:** 10.1038/srep27639

**Published:** 2016-07-04

**Authors:** Muge Sarper, Ernesto Cortes, Tyler J. Lieberthal, Armando del Río Hernández

**Affiliations:** 1 Cellular and Molecular Biomechanics Laboratory, Department of Bioengineering, Faculty of Engineering, Imperial College London, South Kensington Campus, London SW7 2AZ, United Kingdom.

## Abstract

The hallmark of pancreatic ductal adenocarcinoma (PDAC) is abundant desmoplasia, which is orchestrated by pancreatic stellate cells (PSCs) and accounts for the majority of the stroma surrounding the tumour. Healthy PSCs are quiescent, but upon activation during disease progression, they adopt a myofibroblast-contractile phenotype and secrete and concomitantly reorganise the stiff extracellular matrix (ECM). Transforming growth factor β (TGF-β) is a potent activator of PSCs, and its activation requires spatiotemporal organisation of cellular and extracellular cues to liberate it from an inactive complex with latent TGF-β binding protein (LTBP). Here we study the mechanical activation of TGF-β by PSCs *in vitro* by investigating LTBP-1 organisation with fibrillar fibronectin and show that all trans-retinoic acid (ATRA), which induces PSC quiescence, down-regulates the ability of PSCs to mechanically organise LTBP-1 and activate TGF-β through a mechanism involving myosin II dependent contractility. Therefore, ATRA inhibits the ability of PSCs to mechanically release active TGF-β, which might otherwise act in an autocrine manner to sustain PSCs in an active state and a tumour-favouring stiff microenvironment.

Pancreatic cancer is a devastating disease with dismal prognosis: it carries an overall 5 year survival rate of 6% and it is the fourth most common cancer related death in Western world^1^. This is due to the aggressive behaviour of the disease: it metastasises to other organs and resists conventional chemotherapy, which remain the main challenges in pancreatic cancer treatment^1^. PDAC is characterised by an abundant desmoplastic response, defined as sustained proliferation and concurrent deposition of altered cancer-supporting ECM by stromal cells, leading to a stiff tumour-favouring microenvironment^2^. This dense desmoplastic PDAC stroma could act as a barrier to chemotherapeutic agents by making cancer cells inaccessible which, in part, explains the resistance to treatment^3^,^4^. Pancreatic cancer-associated PSCs are the major cell type responsible for the characteristic desmoplastic response in PDAC by depositing and remodelling tumour-promoting ECM^5^. Healthy PSCs are quiescent and store retinol (vitamin A) in their cytoplasm; however, during pancreatic cancer progression, they lose retinol droplets and acquire an activated myofibroblast-like contractile phenotype^5^.

Transforming growth factor-β (TGF-β) is a potent cytokine that is integral to many biological events, including its well-established role as an activator of PSCs^5^,^6^,^7^,^8^,^9^,^10^,^11^. When PSCs are exposed to exogenous TGF-β, myofibroblastic markers such as contractility marker α-smooth muscle actin (α-SMA) are activated, and PSCs increase ECM deposition, particularly fibronectin and collagen type-I^12^,^13^,^14^. In the tumour environment, TGF-β can be released from cancer cells, infiltrating immune cells, or atypical acinar cells^15^. Importantly, PSC activation by TGF-β can initiate an autocrine positive feedback loop which perpetuates an activated phenotype^16^,^17^.

TGF-β is secreted as the large latency complex, which contains latency associated peptide (LAP) and latent TGF-β binding protein (LTBP), and it is stored in the ECM in a latent form by LTBP^18^,^19^. TGF-β is activated via a cell-based mechanism through cell-generated tension against matrix resistance and integrin activity, which leads to detachment from LAP and LTBP^9^,^20^,^21^. Fibronectin binds the LTBP N-terminus, and LTBP organises onto fibronectin fibril templates thereby allowing TGF-β to be activated by the cell by releasing it from LTBP^22^,^23^,^24^. It has also been shown that two interrelated mechanical cues, cellular contractility and ECM stiffness, are the key elements used by the cell to apply tension on latent TGF-β^23^,^24^. ATRA, a retinoic acid isomer and the active metabolite of vitamin A, is known to restore the quiescent phenotype in PSCs as well as reduce concomitant ECM synthesis and pancreatic cancer cell invasion^25^,^26^,^27^. It was recently shown to down-regulate cellular stiffness, contractility, and ECM remodelling by PSCs (unpublished data). ATRA has also been shown to promote cell compliance in acute promyelocytic leukemia via cytoskeletal remodelling^28^. Therefore, we hypothesized that the ability of ATRA to promote cell compliance and to reduce ECM remodelling could interfere with the priming and activation TGF-β by PSCs. In this study, we characterised the ability of PSCs to organise LTBP-1 in the ECM and mechanically activate TGF-β. We show that ATRA down regulates this process through a mechanism involving myosin II-dependent contractility and β1 integrins.

## Results

### ATRA treated PSCs fail to organise and align LTBP-1 and fibronectin into fibrils

**Figure 1:**
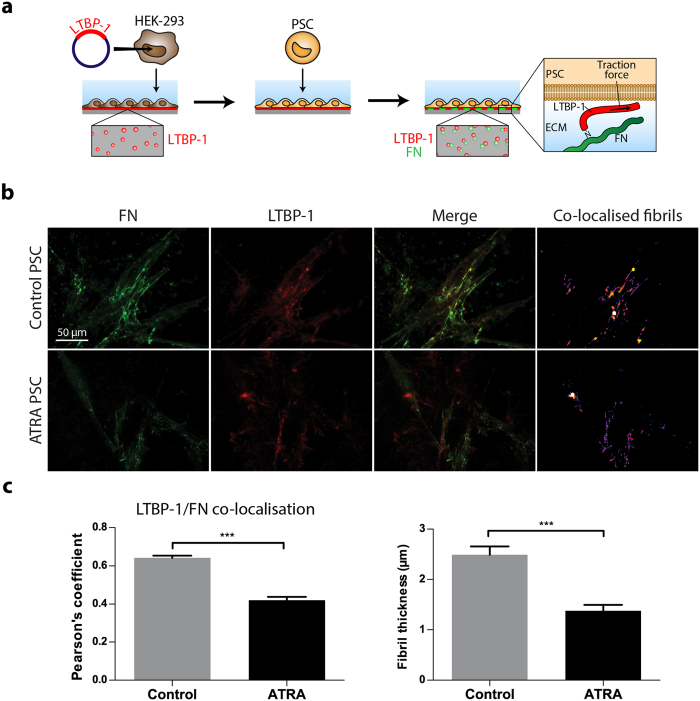
Preparation of LTBP-1 rich ECM and LTBP-1/fibronectin co-localisation by PSCs (**a**) HEK-293 were seeded on coverslips and transfected with LTBP-EGFP. After 7 days of culture, HEK-293 cells were removed with DOC buffer and replaced by PSCs which secrete fibronectin (FN) into the HEK-293-generated ECM. Interaction between LTBP-1 and fibronectin is analysed after 2 days of culture by immunofluorescent co-localisation. (**b**) Colocalisation of LTBP-1 (red) and fibronectin (green) as a result of 10-day treated ATRA (1 μM) PSCs or control PSC incubation. (**c**) Quantification of colocalisation and fibre thickness. Representative for 3 independent experiments;, **p < 0.01, ***p < 0.001, two-tailed Student’s t-test, error bars show means ± SEM.

To study how PSCs organise the ECM under ATRA treatment, we considered their ability to organise LTBP-1 into fibronectin fibrils. The cellular ability to organise LTBP-1 into fibrils has been used before as a standard method to assess TGF-β activation^24^. We first generated an LTBP-1-rich ECM by allowing LTBP-1-transfected HEK-293 cells to deposit ECM on glass coverslips for 7 days (Fig. S1a). The HEK-293 cells were then removed with sodium deoxycholate (DOC) buffer as described in the materials and methods and in Fig. 1a. We used HEK-293 cells because they deposit minimal fibronectin and they were not able to organise LTBP-1 into fibrils (Fig. S1b). After removing HEK-293 cells, untreated PSCs (hereafter control) or 10 day ATRA (1 μM) pre-treated PSCs (hereafter ATRA) were seeded on matrices and cultured for a further 2 days, during which time PSCs secrete and incorporate fibronectin into the ECM. Endogenous expression of LTBP-1 by PSCs was not detectable during 48 hours either on glass coverslips or HEK-293 deposited ECM (Fig. S1c,d). Coverslips were then dual-stained for LTBP-1 and fibronectin. The ImageJ co-localisation plugin coloc 2 was used determine co-localisation of LTBP-1 and fibronectin (Fig. 1a) which quantified the interaction between LTBP-1 and fibronectin. LTBP-1 and fibronectin co-immunostaining showed that in ECM that is remodelled by control PSCs, LTBP-1 can be organised onto fibronectin-positive fibrils (Fig. 1b). In contrast, LTBP-1 remains in patches when deposited by HEK-293 alone (Fig. S1b) as further evidenced by the near zero Pearson correlation coefficient of LTBP-1/fibronectin co-localisation (not shown). Furthermore, LTBP-1 and fibronectin co-localisation was significantly reduced with ATRA treatment compared to vehicle treated PSCs (p < 0.001) (Fig. 1b,c). We then analysed the thickness of the co-localised fibrils as a measure of LTBP-1/fibronectin fibril maturation. The fibril thickness heat-map revealed that LTBP-1 and fibronectin co-localised fibril thickness was significantly reduced with ATRA treatment compared to the control group (p < 0.001) (Fig. 1c). Thus, ATRA reduced LTBP-1 and fibronectin co-localisation and alignment, which is a crucial PSC matrix remodelling behaviour.

### Fibronectin splice variants fibronectin-EDA and fibronectin-EDB are expressed by PSCs, and LTBP-1 alignment requires fibronectin but is unaffected by fibronectin levels in the ECM

**Figure 2:**
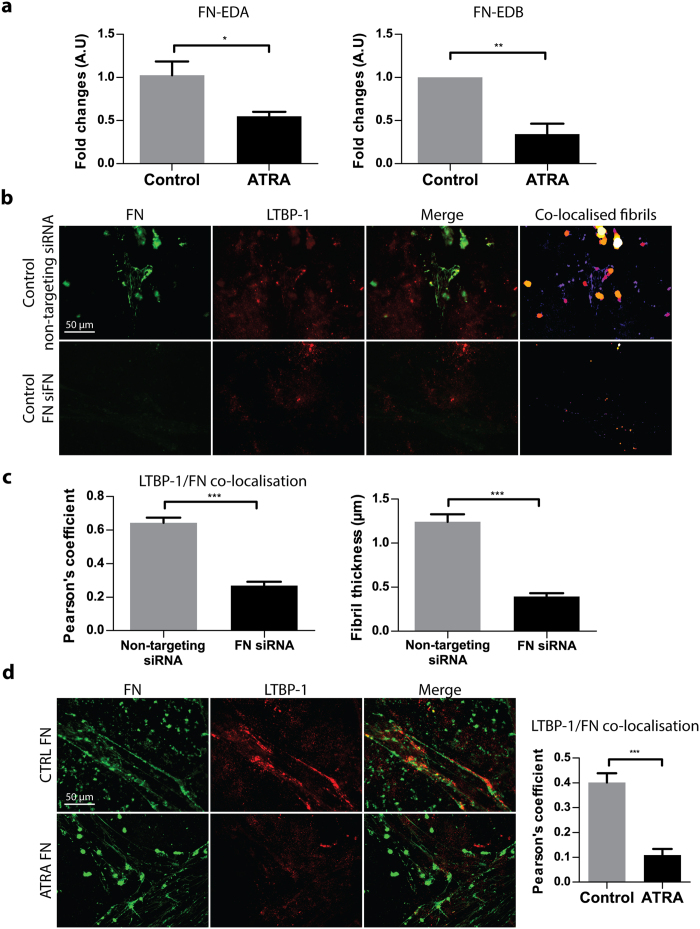
Fibronectin alternative splicing in PSCs under ATRA treatment and effect of fibronectin knock-down and excess fibronectin incubation on fibronectin/LTBP-1 lining (**a**) Q-PCR targeting fibronectin-EDA or fibronectin-EDB mRNA showed a significant decrease in fibronectin-EDA and fibronectin-EDB levels in ATRA treatment group in comparison to control PSC cells. (**b**) Immunostaining images of non-targeting siRNA or fibronectin mRNA targeting siRNA treated control PSCs show that in the absence of fibronectin, LTBP-1 fibril alignment is significantly down-regulated. (**c**) Fibronectin/LTBP-1 co-localisation analysis of control PSCs and ATRA treatment group cultured in the presence of excess fibronectin revealed a significant decrease in ATRA treatment when compared to control. Representative for 3 independent experiments; *p < 0.05, **p < 0.01, ***p < 0.001, two-tailed Student’s t-test, error bars show means ± SEM, A.U. arbitrary units.

Fibronectin is alternatively spliced in wound healing and cancer stroma^29^; for instance, the fibronectin extra domain-A (EDA) isoform is overexpressed in liver tumour vasculature^30^. To understand whether a similar alternative splicing mechanism occurs in PSCs, we examined the expression of fibronectin-EDA, which is the major isoform expressed in breast cancer, and fibronectin-EDB, which has been implicated in pancreatic cancer microenvironment but of an unknown cellular origin^31^. Quantitative PCR targeting fibronectin-EDA or fibronectin-EDB mRNA showed that both variants are expressed by PSCs and expression levels were significantly reduced upon ATRA treatment (p < 0.05, p < 0.01 respectively) (Fig. 2a).

To further dissect whether LTBP-1 fibril formation is dependent on the presence of fibrillar fibronectin, fibronectin expression was knocked down by siRNA (50 nM) in control PSCs prior to culturing the cells for 48 hours on LTBP-1 rich ECM. As a result of fibronectin knockdown, LTBP-1/fibronectin co-localisation and fibril thickness were significantly decreased (Fig. 2b,c) (p < 0.001 for both measurements). This demonstrates that LTBP-1 fibril formation by PSCs is dependent on fibronectin guidance, and in the absence of fibronectin, LTBP-1 is not organised in fibrils.

To exclude the possibility that the reduction in fibronectin/LTBP-1 co-localisation and fibril thickness with ATRA treatment (Fig. 1) might be simply due to differences in expression of extracellular fibronectin (Fig. 2a), control or ATRA treated PSCs were cultured on LTBP-1 rich matrices for 48 hours in the presence of excess fibronectin (40 μg/cm^2^). Despite excess fibronectin addition to culture media, there was a persistent significant decrease in fibronectin/LTBP-1 co-localisation with ATRA treatment (p < 0.001) as observed previously with endogenous fibronectin secretion (Fig. 2c). Therefore, PSC-mediated LTBP-1 fibril organisation onto fibrillar fibronectin depends on the presence of fibronectin but is insensitive to levels of fibronectin in the ECM. Taken together our results indicate that there is a threshold of fibronectin concentration that is needed for LTBP-1/fibronectin alignment by PSCs, but provided this concentration is present, other cellular mechanisms in PSCs control LTBP-1/fibronectin alignment.

### LTBP-1 fibril alignment onto fibrillar fibronectin by PSCs is an actomyosin dependent mechanism

**Figure 3:**
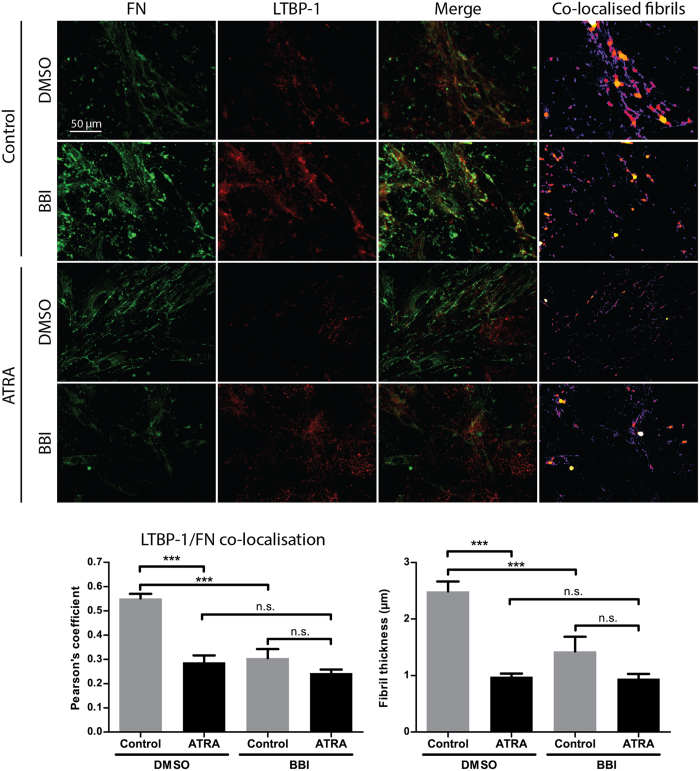
Effect of actomyosin cytoskeleton inhibition on LTBP-1 and fibronectin fibril alignment. Fibronectin/LTBP-1 co-localisation and fibril density analysis of immunofluorescent images of BBI or DMSO only treatment of PSCs showed a significant difference between control and ATRA group in vehicle control condition; however, under BBI treatment control and ATRA groups establish similar fibronectin/LTBP-1 co-localisation levels and fibre thickness. Representative for 3 independent experiments, ***p < 0.001, one-way ANOVA with Tukey’s post-hoc comparison test, error bars show means ± SEM, n.s. not significant.

Because fibronectin/LTBP-1 co-localisation was still significantly down-regulated in ATRA treated PSCs compared to control PSCs even when excess fibronectin is added to culture media, we investigated the role of biomechanical reorganisation of ECM by PSCs *in vitro*. Actomyosin contraction-mediated cellular tension applied to ECM is required for LTBP-1 alignment^23^,^24^,^32^. ATRA has been previously shown to reduce cell stiffness (unpublished data) and cell contractility markers α-SMA and vimentin^25^. Similarly, ATRA reduces expression of cell contractility marker vimentin in PSCs seeded on LTBP-1 rich matrices (Fig. S2). Therefore, to understand the association between PSC contractility and LTBP-1 organisation in our system, control or ATRA treated PSCs were seeded on LTBP-1 rich matrices as previous. PSCs were incubated for 16 hours to allow cell attachment and spreading after which, 20 μM blebbistatin (BBI), which blocks myosin II ATPase activity^33^, or DMSO vehicle was added. Both fibronectin/LTBP-1 co-localisation and the thickness of co-localised fibrils were significantly reduced in untreated PSCs when exposed to BBI (p < 0.001, p < 0.05 respectively) (Fig. 3), indicating that LTBP-1 incorporation onto fibrillar fibronectin is dependent on actomyosin contraction. No significant differences were observed when PSCs were treated with both ATRA and BBI in comparison to ATRA alone (Fig. 3), which indicates that ATRA mainly acts through a reduction of actomyosin contraction. Therefore, inhibition of cell contractility by ATRA or BBI inhibits biomechanical organisation of LTBP-1 and fibronectin.

### PSCs act on LTBP-1 through a β1-integrin mediated mechanism

**Figure 4:**
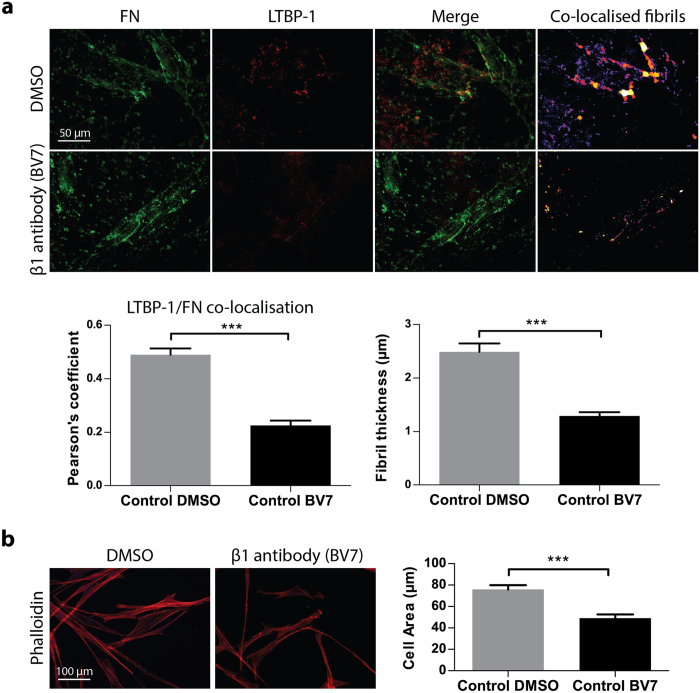
Effect of β1 integrin inhibition on fibronectin/LTBP-1 co-localisation and cell spreading on LTBP-1 (**a**) Immunofluorescent images of control PSCs incubated with β1 integrin blocking antibody (BV7) showed a significant reduction in fibronectin/LTBP-1 co-localisation compared to non-treated group. Co-localised fibre thickness was also reduced in β1 integrin antibody treatment group. (**b**) Phalloidin immunostaining showed a significant reduction in cell area in the control PSCs treated with β1-integrin function blocking antibody when compared to non-treated control PSCs. Representative for 3 independent experiments, 42 cells for DMSO and 35 cells for BV7, ***p < 0.001, two-tailed Student’s t-test, error bars show ± SEM.

LTBP-1 carries an arginyl-glycyl-aspartic acid (RGD) consensus motif, which could act as a recognition site for RGD binding integrins such as the β1 integrin that is responsible for cell-fibronectin adhesion^19^,^34^,^35^. Moreover, cell adhesion to LTBP-1 can be reduced by blocking β1-integrin in myofibroblasts^24^. To determine the importance of β1-integrin activity in LTBP-1 fibril organisation by PSCs, prior to culturing on ECM, the control cell group was incubated with 1 μg/mL β1-integrin function blocking antibody (BV7) for 30 minutes. Control PSCs were then seeded onto LTBP-1 rich matrices and incubated for 48 hours. When β1-integrin was blocked in control PSCs, the fibronectin/LTBP-1 alignment was significantly decreased in comparison to untreated group (p < 0.001) (Fig. 4a). Furthermore, phalloidin staining showed that the cell area occupied by control PSCs was also significantly reduced after β1-integrin inhibition (p < 0.001) (Fig. 4b). These results show that β1-integrin expression by PSCs is a prerequisite for actomyosin-mediated alignment of LTBP-1 and fibronectin.

### ATRA inhibits PSC-mediated liberation of bioactive TGF-β from its latent LTBP-1 complex

**Figure 5:**
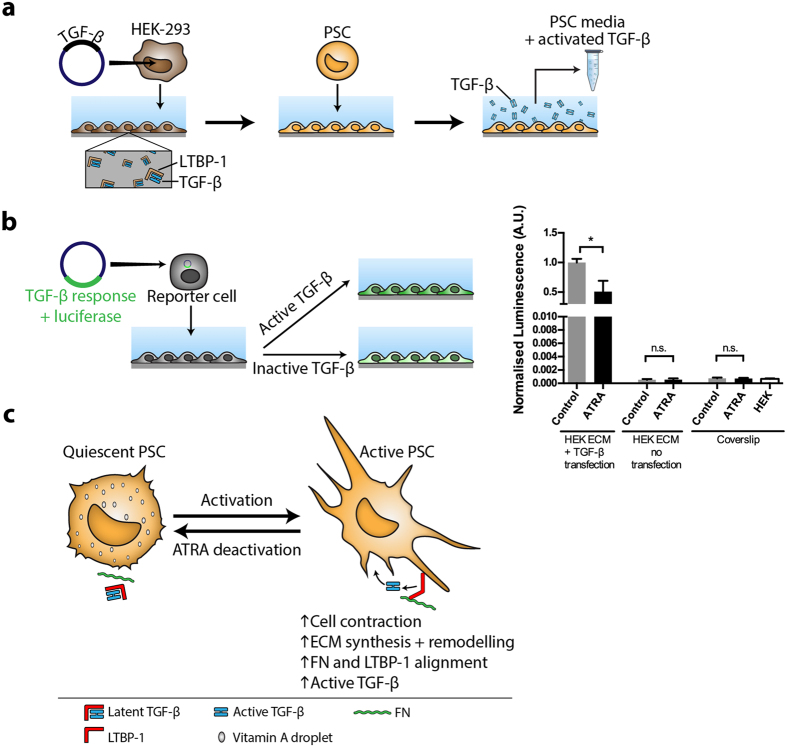
The effect of ATRA treatment on liberation of TGF-β from its LTBP-1 latent form (**a**) Whole TGF-β transfected or non-transfected HEK-293 cells were cultured for 7 days and removed (first arrow). Treated or control PSCs were incubated on matrices for 48 hours during which they can release TGF-β from LTBP-1 into the culture media (second arrow). Active TGF-β containing conditioned culture media was collected. FN, fibronectin. (**b**) Left panel; schematic representation of TGF-β bioassay in which TGF-β reporter cells with a luminescence vector were incubated with active TGF-β containing control or ATRA treated PSC culture media. After ATRA treatment, TGF-β activation by PSCs was significantly reduced (right panel). There were no significant differences in luciferase signal between control and ATRA treated PSCs when cells were seeded on non-transfected HEK-293 ECM or on glass coverslips. The active TGF-β signals were negligible in the following cases: HEK-293 cells or PSCs seeded on glass and PSCs seeded on matrices previously deposited by non-transfected HEK-293. Representative of 3 independent experiments; *p < 0.05, two-tailed Student’s t-test, error bars show means ± SEM, A.U. Arbitrary units. (**c**) Summary schematic illustrating the effects PSC deactivation on ECM remodeling capacity.

Given that ATRA prevented LTBP-1/fibronectin organisation, we investigated whether this was sufficient to inhibit activation of TGF-β from its LTBP-1-bound latent form. To measure the level of TGF-β activated by PSCs, HEK-293 cells were transfected with whole TGF-β coding vector, left to deposit ECM on glass coverslips, then removed as previous. PSCs were then cultured on the ECM for 48 hours in serum-free media. In order to assess baseline active TGF-β production by PSCs during 48 hours, control or ATRA treated PSCs were cultured on non-transfected HEK-293 derived ECM or glass coverslips in serum free media. Conditioned media was collected after the incubation period to quantify the levels of active TGF-β released from the ECM by PSC activity (shown schematically in Fig. 5a). We used a TGF-β reporter cell that carries a luminescent vector fused with TGF-β downstream target PAI-promoter (Fig. 5b); this assay allows quantification of bioactive TGF-β to distinguish it from latent TGF-β and is more physiologically relevant. Reporter cells were incubated for 3 hours in the presence of conditioned media collected from control or ATRA PSCs that were cultured on whole TGF-β matrices. The TGF-β bioassay showed that ATRA treatment significantly reduced active TGF-β levels in the media by 50% when compared to control (p < 0.05) (Fig. 5b), which is consistent with the decrease in fibronectin/LTBP-1 fibril alignment with ATRA treatment. Baseline active TGF-β derived from either control or ATRA treated PSCs was negligible during 48 hours given that media from PSCs grown on non-transfected HEK-293 derived ECM or on glass coverslips produced minimal active TGF-β signal (Fig. 5b). In addition, there was no significant difference in baseline active TGF-β between control or ATRA treated PSCs. Taken together, these findings show that ATRA reduced LTBP-1 alignment and fibronectin/LTBP-1 fibril co-localisation by PSCs which indicates a reduction in ECM remodelling. Concurrently, we showed that ATRA reduces active free TGF-β which could be a result of reduced ECM reorganisation.

## Discussion

In this study we showed that PSC contractility controls concomitant ECM remodelling and active TGF-β release from the ECM. Furthermore, ATRA treatment significantly suppresses the ECM remodelling capacity of PSCs needed to activate TGF-β. We also showed that PSC-mediated LTBP-1 alignment was dependent on fibronectin fibril guidance: in the absence of fibronectin, LTBP-1 fibril assembly was diminished. Yet, the effects of ATRA were not simply a reflection of fibronectin expression levels by PSCs (as shown by the reduction in fibronectin-EDA and fibronectin-EDB expression) because when the system was saturated with excess fibronectin, control PSCs were still able to organise significantly higher levels of LTBP-1 fibrils than the ATRA treated PSC group. Instead, we found that actomyosin cytoskeletal activity was central for LTBP-1/fibronectin alignment because when actomyosin contraction was blocked by BBI, PSCs were unable to organise LTBP-1 into fibronectin fibrils. Treatment of ATRA pre-treated PSCs with BBI did not further reduce LTBP-1/fibronectin co-localisation, thereby confirming that ATRA acts through an actomyosin-dependent mechanism. Therefore, LTBP-1 fibril alignment is a function of cellular contractility and requires fibronectin secretion during TGF-β priming.

The role of α_v_-integrin on LAP is a well-known mechanism of TGF-β activation^9^,^36^; however, the importance of the RGD sequence in human LTBP-1 is yet to be elucidated. Here we found that, in PSCs, β1-integrin activity was needed for LTBP-1 fibril alignment and for cell spreading on LTBP-1 rich ECM in concert with fibronectin secretion. However, blocking β1-integrin activity has a widespread effect, and we cannot exclude the possibility that blocking β1-integrin might affect other adhesion-dependent processes and ECM remodelling behaviour.

Given the paramount relevance of the desmoplastic reaction in the stroma of pancreatic tumours, stromal modulatory drugs have been shown to be a robust strategy which benefits pancreatic cancer treatment by targeting the tumour promoting ability of PSCs, increasing drug delivery to tumour bed, and reducing cancer metastasis^4^,^25^,^37^,^38^. Stromal reprogramming therefore acts by restoring homeostatic conditions in cancer-related microenvironment rather than direct ablation of stromal matrix. ATRA has been shown to restore quiescent phenotype in PSCs by inducing transcriptional reprogramming^25^,^39^. Here we showed that ATRA treatment weakens the capacity of PSCs to activate TGF-β, and TGF-β biological activity is therefore reduced *in vitro* (Fig. 5c). Our findings contribute to previous findings that show ATRA hampers the active myofibroblast PSC phenotype, which is strongly associated with tumour growth and metastasis^25^,^40^. Further work is required to determine if ATRA abrogation of TGF-β activation is sufficient to inhibit secondary effects in a tumour environment such as cancer cell proliferation.

## Materials and Methods

### Cell culture and reagents

HEK-293 cells were cultured in DMEM with high glucose (Sigma-Aldrich, USA) supplemented with 10% FBS (Life Technologies, USA), 2 mM L-glutamine (Sigma-Aldrich, USA), 50 units/ml penicillin and 50 μg/ml streptomycin (Sigma-Aldrich, USA). Primary PSCs were purchased from ScienCell Research Laboratories (Carlsbad, USA) and cultured in DMEM/F-12 HAM (Sigma-Aldrich, USA) with 2% FBS (Life Technologies, USA), 50 units/ml penicillin and 50 μg/ml streptomycin (Sigma-Aldrich, USA), and 5 ml Fungizone (Life Technologies, USA) at 37 °C and 5% CO_2_. Both cell types were tested for contamination.

ATRA (Sigma-Aldrich, USA) was prepared in ethanol and PSCs were treated with 1 μM ATRA to limit cytotoxicity under dim light conditions for 10 days. ATRA treatment produced quiescence markers such as retinol droplets as described previously^25^. The control PSC group was established by treatment with ethanol vehicle-only for 10 days. Media was replenished every 24 hours. Where indicated, cultures were treated with fibronectin or control siRNA (Life Technologies), blebbistatin (Calbiochem, USA) or dimethyl sulfoxide (DMSO), β1-integrin blocking antibody (clone:BV7, ab7168; Abcam, UK) or human plasma fibronectin (FC010) (Millipore, USA).

### HEK-293 transfection

HEK-293 cells were seeded on 13 mm uncoated glass coverslips (11 × 10^4^ cells/coverslip), incubated overnight, and transfected with 0.75 μg pSecTag–LTBP-1–EGFP plasmid (courtesy of Dr Boris Hinz, University of Toronto, Canada) or whole TGF-β plasmid (courtesy of Daniel Rifkin New York University, USA) with JetPrime transfection reagent (Polypus, USA). LTBP-1–EGFP is not loaded with TGF-β whereas whole TGF-β plasmids carry TGF-β and LTBP-1. Media was changed after 4 hours. Transfected HEK-293s were incubated on coverslips for 7 days to deposit ECM.

### ECM de-cellularisation and remodelling

Sodium deoxycholate (DOC) buffer is a water-soluble, ionic detergent commonly used in cell lysis buffers for isolation of cell membrane proteins and lipids. Here we used DOC buffer to disrupt the cell membrane and remove HEK-293 cells from 7 day old ECM. It has been shown previously that the ECM left behind is DOC resistant^24^. DOC was prepared with 150 mM NaCl, 50 mM Tris HCl, 1% NP-40 (v/v) substitute and 0.5% (w/v) sodium DOC (all reagents were purchased from Sigma-Aldrich, USA). Ice cold DOC buffer was added to 7 day HEK-293 cultures on coverslips and incubated at 4 °C for 10 minutes with gentle agitation. The HEK-293 cell debris was washed twice with PBS. Remaining DOC-resistant ECM was incubated with room temperature PBS for 15 minutes. Control or 10-day ATRA treated PSCs (3 × 10^4^ cells) were then seeded on top of de-cellularised matrices and left to remodel ECM under pertinent treatment for 48 hours.

### Gene knock-down

PSCs were reverse transfected with 50 nM fibronectin specific or non-targeting siRNA with JetPrime (Polypus, USA). Prior cell seeded matrices were coated with 200 μl siRNA solution and incubated for 10 minutes, then PSCs were added and cultured.

### TGF-β bioassay

Control or ATRA treated PSCs were starved for 48 hours on whole TGF-β matrices generated from whole TGF-β transfected HEK-293 or on non-transfected HEK-293 as described previously. Conditioned culture media was then collected and stored at −80 °C until needed. The media was concentrated 4 times by centrifugation for 2 minutes at 13000 rpm using centrifugal filter units (Amicon Ultra 0.5 ml centrifugal filters, 10 K, UFC501024, USA). MDAMB231 cell line transfected with plasminogen activator inhibitor-1 (PAI-1) promoter fused with luciferase reporter gene (gifted from Dr Caroline Hill, London Research Institute, London) were used to determine the activity of TGF-β in conditioned media. When the reporter cells are incubated with active TGF-β containing culture media, downstream TGF-β proteins SMAD-2 and SMAD-3 are phosphorylated, form a complex with SMAD-4, and are transported to the nucleus. This complex targets the PAI-1 promoter, which in the reporter cells is fused with luciferase. MDAMB231 reporter cells were seeded in 96 well plates (4 × 10^3^ cells/well) in DMEM supplemented with 10% FBS. MDAMB231 cells were incubated with concentrated PSC culture supernatant or PBS for 3 hours, then washed with PBS and incubated with DMEM + 1% FBS for 16 hours. Luciferase signal was measured with a Luminometer (Fluoroskan Ascent Microplate Fluorometer, Thermo Scientific, UK) with Promega dual luciferase kit according to manufacturer’s instructions.

### Immunofluorescence

PSCs were fixed with 37 °C 4% paraformaldehyde (PFA) for 10 minutes, blocked and permeabilised with 2% BSA and 0.1% Triton X-100 (all Sigma-Aldrich, USA) for 1 hour, incubated with primary antibodies (LTBP-1 MAB388, R&D Systems, USA; fibronectin ab2413, Abcam, UK; vimentin MO725 Dako, Denmark; GFP ab290, Abcam, UK) diluted 1/100 in 2% BSA for 1 hour at room temperature, then washed with PBS and incubated with secondary antibodies (Alexa Fluor 488 anti-rabbit, Rhodamine Red™-X goat anti- mouse, Life Technologies, USA) and phalloidin (Alexa Fluor 546, A22283, Life Technologies, USA) 1/400 diluted in PBS for 45 min in dark. Coverslips were mounted with ProLong Gold Antifade with DAPI (Life Technologies, USA).

### Image acquisition and quantitative analysis

Images were taken with a Motic AE31 trinocular inverted microscope by Motic Images Plus 2.0 software using 40x objective for fibronectin/LTBP-1 analysis and with 20x for phalloidin staining. Co-localisation and fibril density analysis were done with ImageJ (NIH) software by choosing 5 similar-sized (702 × 702 pixels) region of interests (ROI) per image in symmetry. Three images were analysed per condition per experiment. For 3 experiments, n = 45 per condition. The ROIs were then split into red and green channels. The amount of co-localisation of LTBP-1 (red channel) and fibronectin (green channel) was analysed with Coloc2 plug-in by obtaining Pearson’s correlation coefficient (R value) which uses deviation from mean and is not affected from offset^41^. In order to analyse the fibril density co-localisation threshold images were created, converted into binary and analysed with BoneJ plug-in (NIH) by using thickness option. The intensity of vimentin staining was analysed by quantifying the mean fluorescence intensity (MFI) with ImageJ.

### Real-time quantitative polymerase chain reaction (Q-PCR)

Total RNA was extracted from 10-day treated control or ATRA PSCs and reversed transcribed by using a high capacity RNA-to-cDNA kit (Applied Biosystems, USA). Q-PCR was performed with SYBR Green Master Mix (Life Technologies, USA) in a StepOnePlus™ system (Applied Biosystems, USA) using 100 ng cDNA input. Target gene expression was calculated upon normalisation against GAPDH housekeeping gene by using 2^−ΔΔ^CT method. Primers sequences were as follows; fibronectin-EDA forward; 5′-TCCAAGCGGAGAGAGT-3′, reverse; 5′-GTGGGTGTGACCTGAG-3′, fibronectin-EDB forward; 5′-CCACCATTATTGGGTACCGC-3′, reverse; 5′-CGCATGGTGTCTGGACCAATG-3′ and GAPDH forward; 5′ACAGTTGCCATGTAGACC-3′, reverse; 5′-TTTTTGGTTGAGCACAGG-3′.

### Statistical analysis

Control and ATRA treated conditions were compared by two-tailed Student’s *t*-test (GraphPad Prism, San Diego, CA). The p-values less than 0.05 were regarded as significant. For multi group comparisons a one-way ANOVA with Tukey’s post-hoc comparison test was used. A single asterisk indicates *p < 0.05, a double asterisk indicates **p < 0.01, and a triple asterisk indicates ***p < 0.001. Error bars are standard errors of mean (SEM).

## Additional Information

**How to cite this article**: Sarper, M. *et al*. ATRA modulates mechanical activation of TGF-β by pancreatic stellate cells. *Sci. Rep.* **6**, 27639; doi: 10.1038/srep27639 (2016).

## Supplementary Material

Supplementary Information
