# Supplementary material for: ATRA modulates mechanical activation of TGF-β by pancreatic stellate cells
Source: Sci Rep. 2016 Jul 4;6:27639. doi: 10.1038/srep27639 (PMC4931506; doi:10.1038/srep27639)
Supplement: Supplementary Information [file srep27639-s1.pdf]

ATRA modulates mechanical activation of TGF-β by pancreatic stellate cells

Muge Sarper, Ernesto Cortes, Tyler J. Lieberthal, & Armando del Río Hernández*

Cellular and Molecular Biomechanics Laboratory, Department of Bioengineering, Faculty of Engineering, Imperial College London

*Corresponding author: Armando del Río Hernández

Cellular and Molecular Biomechanics Laboratory, Department of Bioengineering, Faculty of Engineering, Imperial College London, South Kensington Campus, London SW7 2AZ, United Kingdom a.del-rio-hernandez@imperial.ac.uk

0044 (0)20 7594 5187

Supplementary Information

Supplementary figure 1

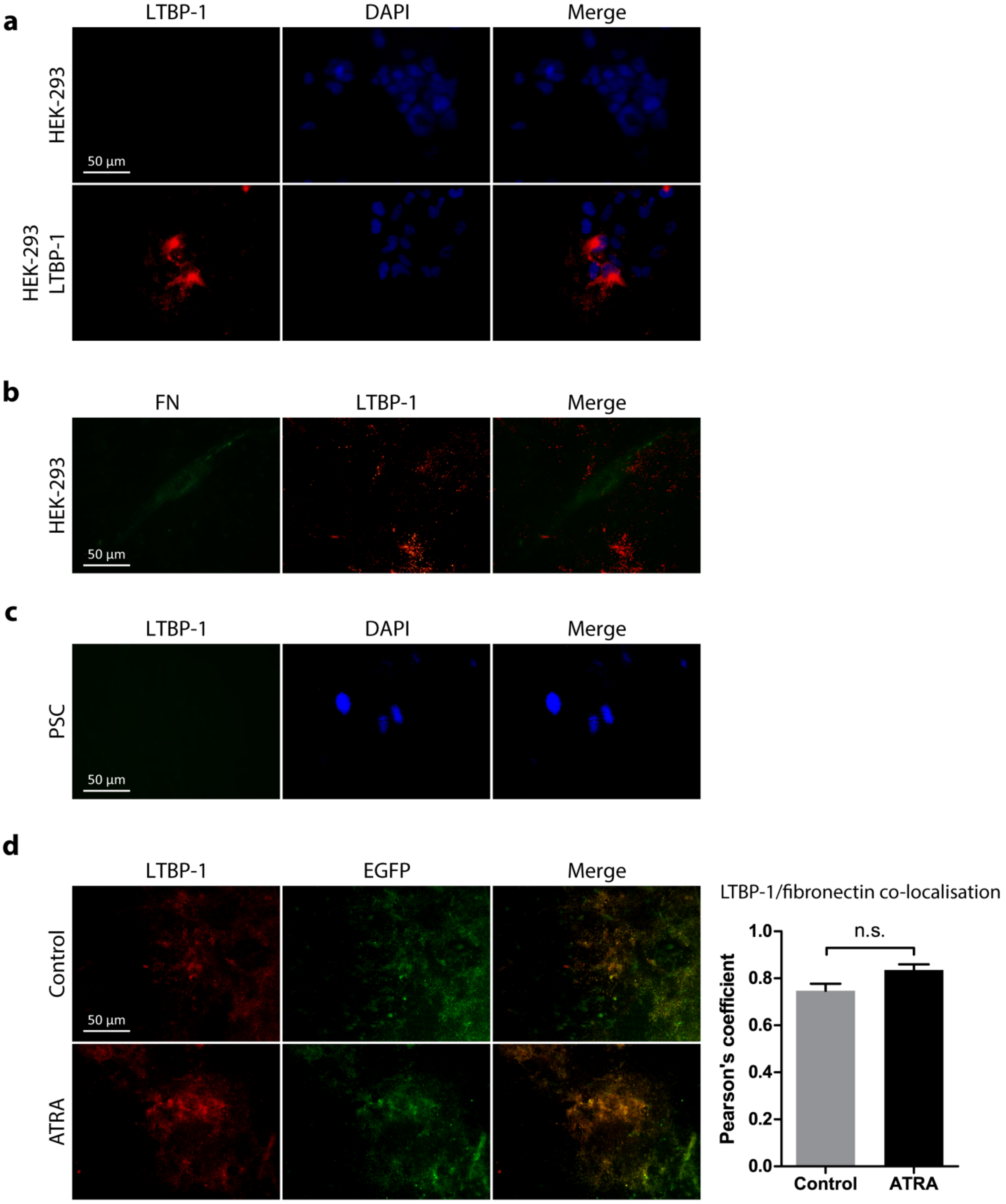

Figure S1. Characterisation of HEK-293 generated ECM matrices. (**a**) Immunofluorescent images show LTBP-1 (red) over-expression in HEK-293 cells. DAPI shows nuclear staining. (**b**) LTBP-1 transfected HEK-293 cells were fluorescently immunostained for fibronectin (green) and LTBP-1 (red) as described in materials and method section. There was no LTBP-1 fibril organisation by HEK293 cells. (**c**) PSCs were seeded on glass coverslips and after 48 hours stained for LTBP-1. LTBP-1 expression by PSCs was found to be minimal. DAPI shows nuclear staining. (**d**) Control or ATRA treated PSCs were seeded on pre-deposited LTBP-1 rich matrices and incubated for 48 hours. Coverslips were then immunostained for EGFP (green) and LTBP-1 (red). Co-localisation analysis indicated that LTBP-1 deposited in the ECM is $EGFP^+$ (HEK-293 derived rather than PSC derived), which by extension indicates a negligible secretion of TGF-β by PSCs during the 48 hour incubation. Representative of 3 independent experiments, Error bars show ± SEM, n.s. = not significant.

Supplementary figure 2

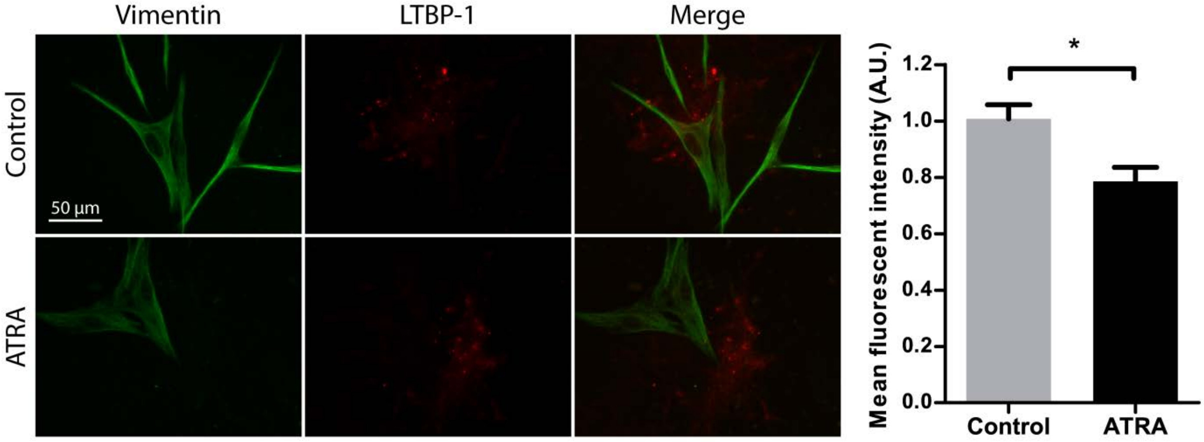

Figure S2. Expression of cell contractility marker vimentin on LTBP-1 matrices. ATRA treated or control PSCs were seeded on LTBP-1 rich matrices for 48 hours and stained for vimentin. ATRA reduced the expression of vimentin, indicating a reduction in a contractile phenotype. Representative of 1 experiment, 28 cells for control and 13 cells for ATRA, *p<0.05, error bar shows ± SEM, A.U. = arbitrary units.
